# Temperature and salinity as key drivers of eggs hatching success in sibling species of the *Contracaecum rudolphii* (s.l.) complex from European waters

**DOI:** 10.1017/S0031182025101108

**Published:** 2026-02

**Authors:** Marialetizia Palomba, Beatrice Belli, Gianpasquale Chiatante, Marta Favero, Daniele Canestrelli, Giuseppe Nascetti, Simonetta Mattiucci

**Affiliations:** 1 Department of Ecological and Biological Sciences, ‘Tuscia University,’ Viale dell’Università, snc, Viterbo, Italy; 2 Department of Health, Well-being, and Environmental Sustainability, ‘Sapienza University of Rome’, Rome, Italy

**Keywords:** abiotic factors, anisakid nematodes, *Contracaecum rudolphii* sp. B, *Contracaecum rudolphii* sp. A, egg hatching, salinity, temperature

## Abstract

Egg hatching is a critical stage in the life cycle of parasitic nematodes and is strongly influenced by abiotic factors. This study investigates, under *in vitro* condition, the effects of temperature (5 °C, 10 °C, 20 °C, 30 °C) and salinity (0–70 psu) on egg hatching success in the two sibling species *Contracaecum rudolphii* sp. A and *C. rudolphii* sp. B, which have been hypothesized to be adapted to brackish/marine and freshwater environments, respectively. Hatching was completely inhibited at 5 °C in both species. At temperature of 10 °C and above, both taxa showed successful hatching with largely overlapping thermal profiles; however, *C. rudolphii* sp. A achieved a marginally significantly higher success, with maximum hatching observed at 30 °C – a value chosen to simulate a potential heatwave scenario. Temperature also influenced developmental timing, with faster hatching occurring at higher temperatures. In contrast, significant marked differences were observed along the salinity gradient: *C. rudolphii* sp. A hatched across a wide range (0–70 psu); while *C. rudolphii* sp. B was restricted to 0–20 psu, with a steep decline above 10 psu. The observed species-specific hatching dynamics, primarily driven by salinity factor, support differential ecological adaptation of the two taxa in their respective aquatic habitats. These findings also provide a basis for predicting parasite responses to environmental change, including rising temperatures and salinity shifts in aquatic ecosystems.

## Graphical abstract

**Figure:**
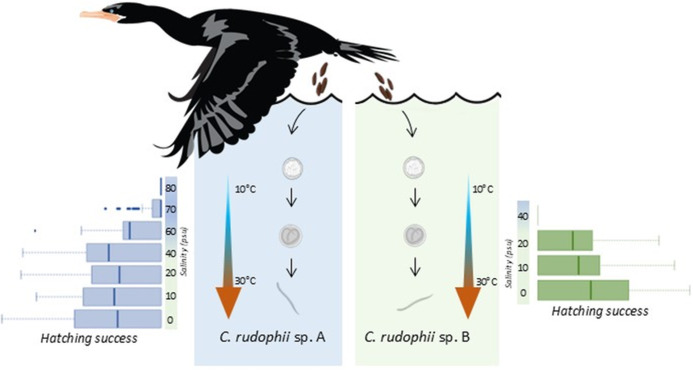

## Introduction

Egg hatching is a critical developmental step in the life cycle of parasitic nematodes, marking the shift from a protected, quiescent embryonic stage to a motile or infective larva (Mkandawire et al. 2022). The eggshell – composed of lipid, chitin, and vitelline layers, and sometimes an additional uterine layer – provides mechanical protection and selective permeability, allowing eggs to remain viable under adverse abiotic conditions for extended periods (Muller 1953; Wharton 1980; Stein and Golden 2018; Lindgren et al. 2020). In aquatic environments, abiotic factors such as temperature, salinity, and oxygen availability strongly influence both hatching success and larval viability (Muller 1953). Hatching represents the first environmentally triggered developmental activation and is highly sensitive to even minor fluctuations in these conditions (Warkentin 2011; Mkandawire et al. 2022). Small changes in temperature or salinity can accelerate, delay or completely inhibit hatching, with cascading effects on larval survival, infectivity, and transmission potential (Born-Torrijos et al. 2014; Montory et al. 2018; Mkandawire et al. 2022). Despite its ecological importance, the effects of abiotic stressors on early developmental stages in aquatic anisakid nematodes are still poorly understood and insufficiently explored.

Avian anisakids of the genus *Contracaecum* constitute a diverse group of heteroxenous nematodes with a cosmopolitan distribution across freshwater, brackish, and marine ecosystems. Their life cycle typically involves copepods or other aquatic invertebrates as intermediate hosts, teleost fish as paratenic hosts, and piscivorous birds as definitive ones (Moravec 2009). *Contracaecum rudolphii* (s.l.) is considered a complex of sibling species that parasitize cormorants of family Phalacrocoracidae in both Boreal and Austral regions (Mattiucci et al. 2002, 2020; Shamsi et al. 2009; Garbin et al. 2011; D’Amelio et al. 2012; Shamsi 2019; Caffara et al. 2023). The life cycle of *C. rudolphii* (s.l.) species complex includes larval embryonation inside the egg, leading to the development of a first-stage larva (L1), which must hatch to initiate transmission. After hatching, the free L2 larva infects a first intermediate host, although this stage remains poorly characterized. Several experimental studies have investigated potential candidates, such as copepods, amphipods and isopods by exposing them to larval stages under controlled condition (Mozgovoy et al. 1965, 1968; Huizinga 1966; Koie 2001; Moravec 2009). Teleost fish serve as paratenic hosts, where third-stage larvae (L3) accumulate and remain infective until transmission to the definitive host, i.e. mainly cormorants.

In European waters, *C. rudolphii* sp. A and *C. rudolphii* sp. B (Bullini et al. 1986; Mattiucci et al. 2002, 2020) provide a valuable model for investigating eco-physiological adaptation in aquatic parasitic nematodes. Although these reproductively isolated taxa share the same definitive host, i.e. the great cormorant, *Phalacrocorax carbo sinensis* (Mattiucci et al. 2002, 2020; Szostakowska and Fagerholm 2007), they are hypothesized to have a life cycle adapted to different aquatic environments. Specifically, *C. rudolphii* sp. A is predominantly associated with brackish and marine habitats, whereas *C. rudolphii* sp. B is more commonly found in freshwater systems (Mattiucci et al. 2002, 2020). Given their distinct environmental distribution, it can be hypothesized that egg hatching success in the two sibling species may be influenced by abiotic factors such as temperature and salinity. These variables may shape species-specific developmental thresholds, reflecting ecological adaptation. However, no comparative studies have yet assessed how these factors influence hatching in these taxa.

To address this gap, this study aims to investigate the effects of temperature and salinity on egg hatching in the two sibling species of the *C. rudolphii* (s.l.) complex occurring in European waters, by exposing eggs to controlled environmental gradients under *in vitro* conditions, in order to test whether the two species exhibit distinct hatching responses, potentially reflecting their ecological adaptation to different aquatic environments.

## Materials and methods

### Collection and isolation of *C. rudolphii spp.* eggs

Adult nematodes of *C. rudolphii* (s.l.) were collected from the gastrointestinal tracts of two great cormorants found dead in the Latium region (Central Italy) during the winter seasons between 2021 and 2024 – one entangled in fishing nets and the other one stranded along the riverbank. From each cormorant, 10 gravid live female nematodes were selected, rinsed twice in autoclaved natural freshwater, and processed within 48 hours. Approximately, 1000 eggs were carefully extracted from the terminal section of the uterus of each live female.

The eggs were repeatedly washed with natural freshwater (filtered through 0.45 µm membranes and autoclaved) on a 30 µm mesh filter. Eggs from each individual female were then transferred into separate wells of 6-well cell culture plates, each containing the same sterile freshwater. The eggs were stored under sterile conditions at +4 °C for 3 days. Molecular identification of each female was carried out during this storage period (details in the next paragraph), after which the eggs were used in the hatching experiments. Eggs of both sibling species were tested simultaneously. After hatching, L2 larvae were counted and monitored daily for the following days. When the first mortalities were observed, the experiment was terminated.

### Molecular identification of *C. rudolphii (s.l.)*

Total genomic DNA from ∼2 mg of each female was extracted using Quick-gDNA Miniprep Kit (ZYMO RESEARCH) following the standard manufacturer recommended protocol. The ITS region of rDNA, including the first internal transcribed spacer (ITS-1), the 5.8S gene, the second transcribed spacer (ITS-2), and ∼70 nucleotides of the 28S gene, was amplified using the primers NC5 (forward; 5′-GTAGGTGAACCTGCGGAAGGATCATT-3′) and NC2 (reverse; 5′-TTAGTTTCTTTTCCTCCGCT-3′) (Zhu et al. 1998). Polymerase chain reactions (PCRs) were carried out in a 15 µL volume containing 0.3 µL of each primer 10 mM, 2.5 µL of MgCl2 25 mM (Promega), 15 µL of 5 × buffer (Promega), 0.3 µL of DMSO, 0.3 µL of dNTPs 10 mM (Promega), 0.3 µL (5 U/μL) of Go-Taq Polymerase (Promega) and 2 µL of total DNA. PCR temperature conditions were the following: 94 °C for 5 min (initial denaturation), followed by 30 cycles at 94 °C for 30 s (denaturation), 55 °C for 30 s (annealing), 72 °C for 30 s (extension) and followed by post-amplification at 72 °C for 5 min. Additionally, the cytochrome c oxidase subunit 2 (*cox*2) locus of the mtDNA was amplified using the primers 211 F (forward; 5′-TTTTCTAGTTATATAGATTGRTTYAT-3′) and 210 R (reverse; 5′-CACCAACTCTTAAAATTATC-3′) (Nadler and Hudspeth 2000; Valentini et al. 2006). PCRs were carried out in a 25 µL volume containing 2 µL of each primer 10 mM, 4 µL of MgCl2 25 mM, 5 µL of 5 × buffer, 2 µL of dNTPs 10 mM, 0.25 µL (5 U/μL) of Go-Taq Polymerase and 3 µL of total DNA. PCR temperature conditions were the following: 94 °C for 3 min, followed by 35 cycles at 94 °C for 30 s, at 46 °C for 1 min, at 72 °C for 90 s, and followed by post-amplification at 72 °C for 10 min.

The successful PCR products were purified, and Sanger sequenced on an Automated Capillary Electrophoresis Sequencer 3730 DNA Analyzer (Applied Biosystems), using the BigDye® Terminator v3.1 Cycle Sequencing Kit (Life Technologies). The obtained sequences were analysed, edited, and assembled by Sequence Matrix v. 1.7.839 and compared with those available in GenBank using BLASTn (Morgulis et al. 2008).

### In vitro exposure to temperature and salinity gradients

Eggs of *C. rudolphii* (s.l.) were exposed to a salinity gradient (0, 10, 20, 40, 60, 70 and 80 practical salinity units [psu]), obtained by dissolving analytical-grade sodium chloride (NaCl) in autoclaved, filtered natural freshwater. This range was selected to encompass the ecological variability of salinity conditions observed in the natural habitats, where the life cycle of the two species of *C. rudolphii* (s.l.) takes place, i.e. from freshwater to hypersaline coastal environments (Mattiucci et al. 2020). Each salinity level was tested under four constant temperature regimes (5 °C, 10 °C, 20 °C and 30 °C), reflecting the average thermal conditions typical of seasonal variation. The upper extreme (30 °C) was included to simulate a heatwave scenario, which is common in hypersaline environments during summer periods. All temperature treatments were maintained in climate-controlled chambers to ensure constant and reproducible conditions.

Hatching experiments were carried out using eggs collected from eight gravid females of *C. rudolphii* (s.l.), four identified as *C. rudolphii* sp. A and four as *C. rudolphii* sp. B. For each species, two females originated from one individual cormorant host and two from another one. Approximately, 5000 eggs were collected from each female and distributed into experimental wells in aliquots of ∼50 eggs per replicate. Experiments were conducted across a full matrix of temperature (30 °C, 20 °C, 10 °C, 5 °C) and salinity (0, 10, 20, 40, 60, 70, 80 psu) conditions. For each temperature–salinity combination, three independent biological replicates *per* female were set up, resulting in a total of 12 replicates *per* condition per species (i.e. 3 replicates × 4 females).

### Egg hatching success

Daily observations of hatching success and timing were conducted over a 15-day incubation period using a Leica M205 stereomicroscope. Hatching was defined as the complete emergence of the larva from the eggshell (Dziekońska-Rynko and Rokicki 2007). Embryonic development and larval formation were assessed based on morphological features according to Moravec (2009). In detail, larvae were considered hatched only upon reaching the second larval stage (L2), characterized by active movement, a slender body with dense mid-body granulation, and the presence of a loosened second-stage cuticle at both ends of the larval body, as described by Moravec (2009). Hatching success was assessed by directly counting eggs and live larvae in the wells, which were placed on transparent plastic film marked with 1 mm × 1 mm squares and examined under the stereomicroscope. Observations began on the first day of incubation and were conducted daily. Once hatching was first detected, counts were carried out until no further increase in the number of hatched larvae was detected. For each count, four adjacent millimetre squares were randomly selected across the grid, and the total number of individuals within these squares was used to extrapolate to the whole area, following the method described by Højgaard (1998). The count was repeated independently by the same operator at least twice, and the variation between repeated counts was consistently <5%, confirming the robustness of the measurements.

### Statistical analysis

For each replicate, hatching success was calculated as the proportion of hatched L2 larvae relative to the total number of larvae initially placed in the dish (mean 50 ± 3; range 45–55), and hatching success was expressed as the percentage of hatched L2 larvae relative to this initial count. Differences in hatching success between *C. rudolphii* sp. A and sp. B under different incubation temperatures and salinities were assessed using mixed Beta regression model, which is appropriate when the response variable is continuous and bounded by 0 and 1 (Kieschnick and McCullough 2003; Ferrari and Cribari-Neto 2004). Replicate was included as a random factor to account for variability among replicates. In addition, to test the combined effects of temperature and salinity on egg hatching dynamics over time, we built two mixed Beta regression models, one for each species. Specifically, hatching success was used as the dependent variable, while the interaction day × salinity × temperature was included as the independent variable. Model performance was evaluated by calculating the correlation between predicted and observed values, as well as the marginal and conditional *R^2^* (Nakagawa and Schielzeth 2013). All statistical analyses were performed using R version 4.2.2 (R Core Team 2022) and the package *glmmTMB* (Brooks et al. 2017).

## Results

### Molecular identification of *C. rudolphii spp.*

A tissue fragment from each of the 20 adult females was genetically identified by sequence analysis of the ITS region of rDNA and the mitochondrial *cox*2 gene. In the first cormorant (entangled in fishing nets), 7 females were identified as *C. rudolphii* sp. A and 3 as *C. rudolphii* sp. B. In the second cormorant (stranded along the riverbank), 8 females were identified as *C. rudolphii* sp. B and 2 as *C. rudolphii* sp. A. Overall, 12 specimens were identified as *C. rudolphii* sp. A and 8 as *C. rudolphii* sp. B, showing 99–100% sequence identity with reference ITS and *cox*2 sequences previously deposited in GenBank for *C. rudolphii* sp. A and *C. rudolphii* sp. B (accession numbers: OR263224-OR236202 for ITS, OR854803-OR269668 for *cox*2). The sequences generated in this study were deposited in GenBank under accession numbers PV990952 (*cox*2) and PV982888 (ITS) for *C. rudolphii* sp. A and PV990953 (*cox*2) and PV982889 (ITS) for *C. rudolphii* sp. B.

### Temperature and hatching success

**Figure 1.**
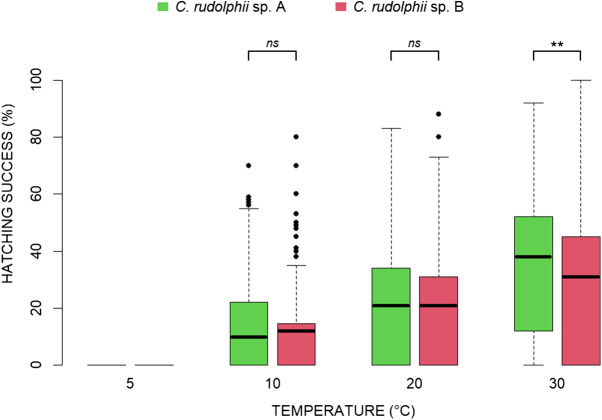
Box plots of hatching success (%) for *C. rudolphii* sp. A (green) and *C. rudolphii* sp. B (red) at four incubation temperatures. Error bars represent standard deviation values. Asterisk indicates statistical significance range: *P < 0.05, **P < 0.001, *ns* = not significant. The thick line within each box represents the mean value.

The effect of temperature on egg hatching success in *C. rudolphii* sp. A and sp. B is shown in Figure 1. No hatching was observed at 5 °C in either species. At 10 °C, both taxa showed limited hatching, with mean success rates of 9.6% for *C. rudolphii* sp. A and 11.6% for *C. rudolphii* sp. B. The difference between species at this temperature was statistically nearly significant (intercept = 0.098 ± 0.014; β = 0.018 ± 0.01; *P* = 0.062) (Figure 1). At 20 °C, hatching success increased moderately in both species, with mean values of approximately 21% for both *C. rudolphii* sp. A and *C. rudolphii* sp. B. However, the interspecific difference at this temperature was not statistically significant (intercept = 0.211 ± 0.02; β = 0.003 ± 0.01; *P* = 0.822). Finally, at 30 °C, both species exhibited their highest hatching performance, with *C. rudolphii* sp. A reaching a mean success rate of 37.6%, compared to 31.3% in *C. rudolphii* sp. B, with a statistically significant difference (intercept = 0.380 ± 0.02; β = −0.067 ± 0.02; *P* < 0.001) (Figure 1).

### Salinity and hatching success

**Figure 2.**
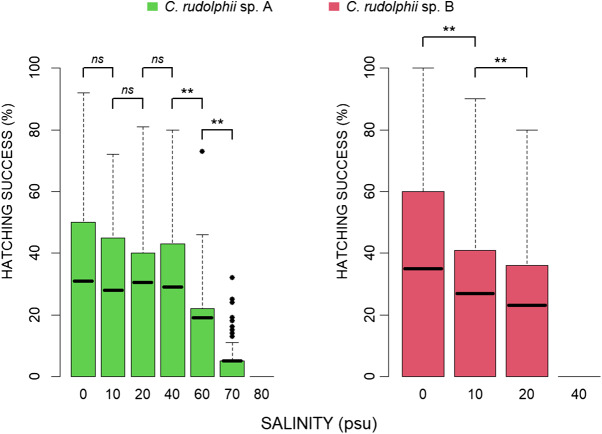
Bar plots of hatching success (%) of *C. rudolphii* sp. A (green) and *C. rudolphii* sp. B (red) at different salinity levels. Error bars represent standard deviation values. Asterisk indicates statistical significance range: *P < 0.05, **P < 0.001, *ns* = not significant. The thick line within each box represents the mean value.

The effect of salinity on egg hatching success in *C. rudolphii* sp. A and *C. rudolphii* sp. B is shown in Figure 2. L2 larvae of C*. rudolphii* sp. A hatched successfully up to 40 psu, with a significant decline at 60 psu and no hatching observed at 80 psu (Figure 2). Hatching success was comparable between 0 and 40 psu, with mean values of ∼30%, and no significant differences observed (intercept = 0.309 ± 0.02; β = −0.022 ± 0.01; *P* = 0.091). However, it decreased significantly at higher salinities: 18.9% at 60 psu (intercept = 0.287 ± 0.02; *β* = −0.083 ± 0.02; *P* < 0.001) and 5.4% at 70 psu (intercept = 0.204 ± 0.02; β = −0.135 ± 0.02; *P* < 0.001). No hatching occurred for *C. rudolphii* sp. A at 80 psu. In contrast, *C. rudolphii* sp. B exhibited successful hatching only up to 20 psu. The highest success was recorded at 0 psu (35.2%), followed by a progressive and statistically significant decline at both 10 psu (27.2%; intercept = 0.353 ± 0.03; β = −0.080 ± 0.01; *P* < 0.001) and 20 psu (23.2%; intercept = 0.272 ± 0.03; β = −0.039 ± 0.01; *P* < 0.001). No hatching occurred for *C. rudolphii* sp. B at 40 psu or above.

### Combined effects of temperature and salinity over time

**Figure 3.**
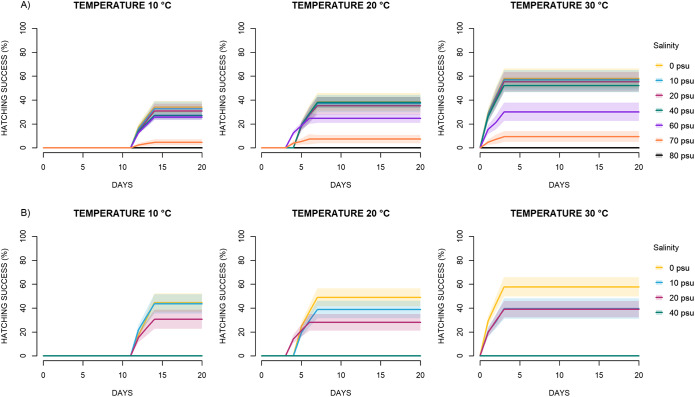
Observed hatching success (%) of *C. rudolphii* sp. A (A) and *C. rudolphii* sp. B (B) under three incubation temperatures (10 °C, 20 °C, 30 °C) and different salinity levels (0–80 psu), over time.

**Figure 4.**
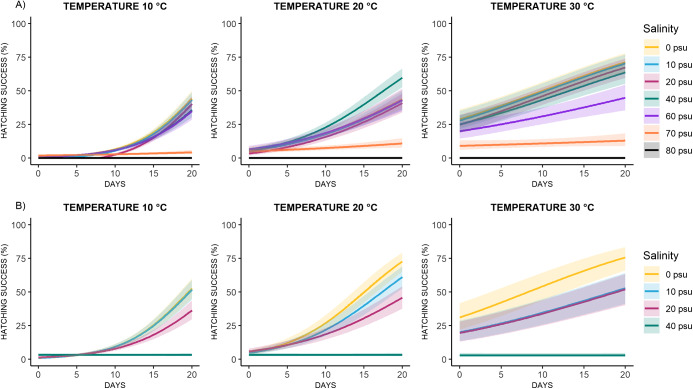
Predicted hatching success (%) of *C. rudolphii* sp. A (A) and *C. rudolphii* sp. B (B) under three incubation temperatures (10 °C, 20 °C, 30 °C) and different salinity levels (0–80 psu), over time. Shaded areas indicate 95% confidence intervals.

The combined effects of temperature and salinity on egg hatching dynamics over time are shown in Figure 3. Both species exhibited similar hatching timelines. At 10 °C, L2 larvae emerged between days 12 and 14; at 20 °C, hatching occurred between days 4 and 7; and at 30 °C, larvae began to emerge as early as days 1–3. In all temperature conditions, hatching success reached a plateau within a few days after the onset of hatching (Figure 3). Model analysis showed that in *C. rudolphii* sp. A, hatching success increased significantly at all temperatures and salinity levels, except at 70 psu, where it decreased significantly at 10 °C and showed no significant effect at 20 °C and 30 °C (Table 1, Figure 4). In *C. rudolphii* sp. B, hatching success increased significantly between 0 and 20 psu across all temperatures (Table 2, Figure 4), but decreased significantly at 40 psu under all temperature conditions.

**Table 1.** Mixed-Beta regression models investigating the effect of salinity (sal0 = 0 psu, sal10 = 10 psu, sal20 = 20 psu, sal40 = 40 psu, sal60 = 60 psu, sal70 = 70 psu, sal80 = 80 psu) on egg hatching success of *C. rudolphii* sp. A over time at 10 (temp10), 20 (temp20), and 30 (temp30) °C. Estimates, standard errors (SE), 95% confidence intervals (LCI, UCI), significance (*P*), correlation between predicted and observed values, and marginal (*R^2^_m_*) and conditional (*R^2^_c_*) *R^2^* are reported

Covariate	Estimate	SE	LCL	UCL	*P*
Intercept	0.101	0.019	–	–	–
Day × temp10 × sal0	0.009	0.001	0.006	0.011	<0.001
Day × temp10 × sal10	0.008	0.001	0.006	0.010	<0.001
Day × temp 10 × sal20	0.006	0.001	0.004	0.008	<0.001
Day × temp10 × sal40	0.005	0.001	0.003	0.008	<0.001
Day × temp10 × sal60	0.006	0.001	0.003	0.009	<0.001
Day × temp10 × sal70	−0.004	0.001	−0.007	−0.001	0.009
Day × temp10 × sal80	0.001	0.001	−0.001	0.003	0.245
Day × temp20 × sal0	0.016	0.001	0.014	0.019	<0.001
Day × temp20 × sal10	0.015	0.001	0.013	0.017	<0.001
Day × temp20 × sal20	0.018	0.001	0.016	0.020	<0.001
Day × temp20 × sal40	0.019	0.001	0.016	0.021	<0.001
Day × temp20 × sal60	0.011	0.001	0.008	0.015	<0.001
Day × temp20 × sal70	−0.001	0.001	−0.004	0.002	0.547
Day × temp20 × sal80	0.000	0.001	−0.002	0.002	0.621
Day × temp30 × sal0	0.035	0.001	0.033	0.037	<0.001
Day × temp30 × sal10	0.033	0.001	0.031	0.035	<0.001
Day × temp30 × sal20	0.034	0.001	0.032	0.036	<0.001
Day × temp30 × sal40	0.031	0.001	0.028	0.033	<0.001
Day × temp30 × sal60	0.016	0.001	0.013	0.019	<0.001
Day × temp30 × sal70	0.001	0.001	−0.002	0.004	0.533
Day × temp30 × sal80	−0.001	0.001	−0.003	0.001	0.496

*Random effect*: replicate; variance = 0.002, SD = 0.051.

Predicted *vs* observed values: *r* = 0.700, *P* < 0.001. *R^2^_m_* = 0.43, *R^2^_c_* = 0.48.

**Table 2.** Mixed-Beta regression models investigating the effect of salinity (sal0 = 0 psu, sal10 = 10 psu, sal20 = 20 psu, sal40 = 40 psu) on egg hatching success of C. Rudolphii sp. B over time at 10 (temp10), 20 (temp20) and 30 (temp30) °C. Estimates, standard errors (SE), 95% confidence intervals (LCI, UCI), significance (*P*), correlation between predicted and observed values, and marginal (*R^2^_m_*) and conditional (*R^2^_c_*) *R^2^* are reported

Covariate	Estimate	SE	LCI	UCI	*P*
Intercept	0.070	0.035	–	–	–
Day × temp10 × sal0	0.015	0.001	0.013	0.018	< 0.001
Day × temp10 × sal10	0.015	0.001	0.013	0.018	< 0.001
Day × temp10 × sal20	0.009	0.001	0.007	0.011	< 0.001
Day × temp10 × sal40	−0.005	0.001	−0.007	−0.003	< 0.001
Day × temp20 × sal0	0.028	0.001	0.026	0.031	< 0.001
Day × temp20 × sal10	0.021	0.001	0.019	0.024	< 0.001
Day × temp20 × sal20	0.015	0.001	0.012	0.017	< 0.001
Day × temp20 × sal40	−0.005	0.001	−0.007	−0.003	< 0.001
Day × temp30 × sal0	0.037	0.001	0.035	0.039	< 0.001
Day × temp30 × sal10	0.024	0.001	0.022	0.026	< 0.001
Day × temp30 × sal20	0.023	0.001	0.021	0.026	< 0.001
Day × temp30 × sal40	−0.005	0.001	−0.007	−0.003	< 0.001

*Random effect*: replicate; variance = 0.010, SD = 0.102.

Predicted *vs* observed values: *r* = 0.783, *P* < 0.001. *R^2^_m_* = 0.46, *R^2^_c_* = 0.61.

In all the experimental conditions where hatching was observed, L2 larvae of both species displayed active movement and were consistently attached to the bottom of the wells. For all experimental conditions, larvae were monitored and counted for 3 days after hatching. During this period, the number of active larvae remained stable, with variations ≤5 individuals per day. A decline, with the first mortalities, was observed only after day 3, at which point the experiments were stopped.

## Discussion

This study provides the first experimental evidence of species-specific hatching responses to temperature and salinity gradients in the sympatric anisakid nematodes *C. rudolphii* sp. A and *C. rudolphii* sp. B. Previous research on *C. rudolphii* (s.l.) reported hatching in seawater at 15 °C and 20 °C (Bartlett 1996). However, in that paper, the two sibling species had not yet been disclosed; therefore, no species-specific differences in hatching dynamics were evidenced.

The results here presented confirm that temperature strongly influences embryonic development and hatching success in *C. rudolphii* (s.l.). Temperature does not appear to be a limiting factor differentiating the two taxa; indeed, both *C. rudolphii* sp. A and *C. rudolphii* sp. B successfully hatched at 10 °C and above, with comparable performance at 20 °C and 30 °C. However, notably, *C. rudolphii* sp. A consistently exhibited slightly higher hatching rates, suggesting a marginal advantage under both cold and warm conditions. Nevertheless, the overall thermal response profiles largely overlapping between the two species (Figure 3). Complete inhibition of development occurred at 5 °C in both taxa, indicating this value as a lower thermal threshold. Comparable thermal tolerance patterns have been observed in other anisakid nematodes. For instance, *Anisakis simplex* (s.s.) and *A. pegreffii* hatched within the range of 3–25 °C and 3–27 °C, respectively, with the latter showing greater tolerance to higher temperatures (Gomes et al. 2023). Similarly, in the digenean *Schistosoma mansoni*, development accelerates above 30 °C but fails beyond this threshold, highlighting the complex and non-linear effects of heat stress on parasite transmission (Pflüger 1980). In the present study, temperature also significantly influenced the timing of larval emergence. At 10 °C, hatching occurred between days 12 and 14; at 20 °C, between days 4 and 7; and at 30 °C, as early as days 1 to 3, confirming a clear acceleration of development with increasing temperature (Figure 3). While high temperatures appear to enhance hatching efficiency and speed, our study did not assess larval survival or infectivity post-hatching, which remains an open question. To evaluate larval viability and competence, experimental infections in suitable intermediate hosts would be necessary. A noteworthy observation during the study was the active swimming and bottom-attachment behaviour of newly hatched L2 larvae in both species. This behaviour suggests that benthic or epibenthic invertebrates may serve as first intermediate hosts in natural environments. This supports earlier hypotheses proposing copepods and/or amphipods as potential initial hosts (Huizinga 1966; Moravec 2009).

In contrast to temperature, salinity emerged as a key ecological factor differentiating the two species. *Contracaecum rudolphii* sp. A hatched successfully up to 70 psu, confirming the parasite’s broad environmental tolerance and its euryhaline adaptation. Conversely, *C. rudolphii* sp. B was restricted to a narrower range (0–20 psu), with hatching success declining sharply above 10 psu and being completely inhibited at higher concentrations. This marked asymmetry suggests that *C. rudolphii* sp. B is specialized for freshwater environments (e.g. lakes and rivers), while *C. rudolphii* sp. A is adapted to variable salinity conditions, including brackish and marine habitats.

Therefore, salinity acts as an ecological driver during early development, contributing to the differential distribution and abundance of these sibling taxa and shaping their life-cycle dynamics. The congruence between experimental tolerance data and field-based host-parasite associations strongly supports the hypothesis of ecological segregation and local adaptation between *C. rudolphii* sp. A and *C. rudolphii* sp. B.

In agreement with these findings, L3 larvae of *C. rudolphii* sp. A significantly prevail in fish from brackish and coastal environments (Mattiucci et al. 2020); whereas *C. rudolphii* sp. B is predominantly found in freshwater fish (e.g. Szostakowska and Fagerholm 2007; Culurgioni et al. 2014; Mattiucci et al. 2020). Analogously, adult specimens of *C. rudolphii* sp. A are frequently found in cormorants inhabiting marine or brackish areas, while *C. rudolphii* sp. B occurs more frequently in cormorants from freshwater ecosystems. This ecological partitioning is further supported by the hypothesis proposed by Marion (1995), which suggests that cormorants exhibit distinct feeding preferences depending on their natal environment: individuals born and raised in freshwater tend to forage primarily in freshwater habitats, while those from marine or brackish origins show a preference for saline environments. These differentiated foraging habits likely drive the distinct parasite assemblages observed in cormorant populations, reinforcing the ecological segregation between the two *C. rudolphii* taxa. This pattern has been documented across several European regions. In Italy, *C. rudolphii* sp. A has been recorded in cormorants from salt marshes and brackish coastal habitats (Mattiucci et al. 2002, 2020; Amor et al. 2020; Carmeño et al. 2022; Cammilleri et al. 2023), with similar findings reported from Spain (Roca-Geronès et al. 2023). Conversely, *C. rudolphii* sp. B has consistently been identified in cormorants from freshwater lakes and rivers, such as Poland (Szostakowska and Fagerholm 2007) and again in Italy (Amor et al. 2020; Mattiucci et al. 2020; Caffara et al. 2023).

## Conclusions

This study provides the first experimental evidence of species-specific hatching responses to water temperature and salinity in the two sibling species *C. rudolphii* sp. A and *C. rudolphii* sp. B, which occur sympatrically and syntopically in the great cormorant, *Ph. carbo sinensis* as their definitive host. While temperature significantly influences embryonic development and hatching success in both taxa, it does not appear to play role in differentiating their ecological preferences. In contrast, salinity results as a key driver shaping species-specific hatching success, highlighting marked differences in environmental tolerance: *C. rudolphii* sp. A exhibited a broad euryhaline capacity, whereas *C. rudolphii* sp. B showed a preference for freshwater conditions.

In conclusion, even when co-infecting the same host, the eggs of these two species – expelled via cormorant faeces – are exposed to abiotic features which are responsible for the hatching success. This pattern greatly contributes to the ecological segregation of these reproductively isolated species, as observed across different aquatic ecosystems.

These findings not only elucidate important aspects of *C. rudolphii* spp. ecology, but also underscore the relevance of this parasite group in the context of ongoing climate change – particularly in coastal and brackish habitats where temperature and salinity fluctuations are expected to intensify. Given that anisakid presence and density may serve as indicators of environmental change (Palomba et al. 2023), species within *C. rudolphii* (s.l.) could also act as valuable sentinels for monitoring ecosystem responses to climate-driven shifts.
